# Multimodal imaging in retinitis pigmentosa related to the *EYS* gene

**DOI:** 10.5935/0004-2749.2024-0104

**Published:** 2024-10-31

**Authors:** Ester Abigail da Silva Martins, Gabriela Doná Rodrigues, Kendy Junior Ivama, Mariana Costa Pereira, Caio Henrique Marques Texeira, Rebeca Azevedo Souza Amaral, Juliana Maria Ferraz Sallum

**Affiliations:** 1 Hospital São Paulo, Escola Paulista de Medicina, Universidade Federal de São Paulo, São Paulo, SP, Brazil

**Keywords:** Retinal diseases/diagnostic imaging, Retinitis pigmentosa/genetics, Retinal degeneration, Eye proteins/genetics, Eye diseases, hereditary/genetics, Genes, recessive, Phenotype, Multimodal imaging, Tomography, optical coherence/methods, Fluorescein angiography, Genetic predisposition to the disease

## Abstract

**Purpose:**

This study aimed to characterize retinitis pigmentosa associated with the *eyes shut homolog* gene, which causes hereditary retinal degeneration.

**Methods:**

The anatomical and functional findings of retinitis pigmentosa in patients with variants of the *eyes shut homolog* gene were characterized and compared using multimodal imaging and genetic analysis of the variants. Clinical data such as visual acuity, lens status, and refraction were obtained from medical records. Patients underwent an ophthalmic examination, including static visual field, microperimetry, optical coherence tomography, fundus autofluorescence, and fundus photography.

**Results:**

Twenty-two patients were included in the study. Several anatomical and functional characteristics of retinitis pigmentosa-*eyes shut homolog* were identified, including the presence of cataracts, cystoid macular edema, epiretinal membrane, and a tubular visual field. Genetic results revealed 26 distinct variants in the cohort, with 7 novel variants not previously documented or reported in the scientific literature or databases.

**Conclusion:**

The findings demonstrate that *eyes shut homolog-*retinitis pigmentosa manifests in specific patterns, starting in adolescence with mild progression and advancing with age. The integration of multimodal imaging and genetic analysis has provided a detailed understanding of the anatomical and functional features of retinitis pigmentosa-*eyes shut homolog*. Seven novel variants of the *eyes shut homolog* gene have been identified. These findings enhance the understanding of *eyes shut homolog*-related retinitis pigmentosa characteristics of by detailing the spectrum of mutations in this gene within the Brazilian population.

## INTRODUCTION

Retinitis pigmentosa (RP) is a progressive retina degeneration disease^(1)^. Its worldwide prevalence is estimated to be 1:4,000 individuals^(2)^. Although a rare disease, RP is the main cause of adult inherited blindness, affecting >1 million people worldwide^(3)^.

RP is also considered an example of genetic heterogeneity, as the typical fundus alteration of RP can be observed in different mutations of different genes^(4)^. More than 80 genes have been identified as causing syndromic and nonsyndromic RP, and the *eyes shut homolog* (*EYS*) gene is related to nonsyndromic RP^(5)^. In some cases, fundus features that resemble RP may appear^(6)^. This occurs when some disorders, hereditary or not, simulate the phenotypic characteristics of RP, which arise due to a stimulus generated by trauma or inflammation and may vary due to hereditary constitution, duration of exposure, and stage of development during exposure^(7)^. This effect is called phenocopy, which is defined as a phenotypic trait or disease that resembles the trait expressed by a particular genotype but in an individual who is not a carrier of that genotype.

In 2008, Abd El-Aziz et al.^(8)^ reported for the first time that the *EYS* gene was identified at the *RP25* locus on chromosome 6q12. Currently, the function of the protein encoded by the *EYS* gene may play a role in retinal morphogenesis, architecture, and ciliary transport^(9)^. There are >4,687 reported variants of the *EYS* gene in ClinVar, a public database that aggregates information about genomic variations^(10)^. Furthermore, the genetic spectrum, clinical manifestations, disease progression, and phenotype show great variations^(11)^.

Multimodal images are performed using several imaging modalities. This type of analysis allows better interpretation of tissue functionality and monitoring of disease progression or therapeutic response, as it uses images that assess the region of interest functionally and anatomically^(12)^.

This study aimed to understand the clinical features of RP related to the *EYS* gene, characterize and compare the anatomical and functional findings detected in the examinations, and observe the disease features in individuals of different ages through multimodal images.

## METHODS

This study was approved by the Research Ethics Committee of the Universidade Federal de São Paulo (approval no. 3.006.920). All volunteers agreed to participate in the study by signing an informed consent form.

This observational study included cases from the Instituto de Genética Ocular (São Paulo, Brazil). It was conducted in the Ocular Genetics Sector of the Department of Ophthalmology and Visual Sciences at the *Universidade Federal de São Paulo-Escola Paulista de Medicina (Hospital São Paulo)*. Individuals with clinical findings consistent with RP and molecular diagnosis with two variants in the *EYS* gene were included. Total cataracts or any other opacity that would prevent the capture of fundus images were considered exclusion criteria.

The following data were collected from participant’s medical records at the *Instituto de Genética Ocular:* visual acuity (VA), lens status, refraction, and molecular testing for genetic disorders. To assess the lens status, participants were classified into two groups: patients without cataracts and patients with cataracts and/or who have had cataract surgery in at least one eye. Static visual field (SVF), microperimetry (MP), spectral-domain optical coherence tomography (SD-OCT), fundus autofluorescence (FAF), and fundus photography (FP) were collected only for this study’s purpose, performed in a single visit from August 15 to November 18, 2022. The demographic information of the sample was obtained before the start of the examinations based on a brief anamnesis.

### SVF

To evaluate SVF, the Humphrey HFA II 745 version 57,445.14.0 (Carl Zeiss, Germany) visual perimeter was used. In this study, the 30-2 program and the SITA-Fast strategy were used. The following variables were analyzed: foveal sensitivity threshold and visual field classification, which were performed based on the graph in gray tones. False-negative, false-positive, and fixation loss rates were also observed to analyze whether the results were reliable.

### MP

A Centervue MAIA version 2.5.1 (Italy) microperimeter was used to evaluate retinal sensitivity. The average macular threshold data and fixation stability were collected and evaluated. The examination was conducted in Expert Exam mode in the 4-2 strategy, first evaluating 68 points in the central 20^o^, to observe retinal sensitivity at a greater angle. To verify the difference between the periphery and the center of the fundus, an evaluation of 32 points in the central 10^o^ was performed.

### FP

The Zeiss Visucam 524 (Carl Zeiss Meditec, Germany) device was used for the photographic documentation of the retina. Images were analyzed to verify if they presented the characteristic clinical features of RP. Color images were captured in the following fields: posterior pole, nasal, temporal, superior, and inferior.

### FAF

A confocal scanning ophthalmoscope (Heidelberg retina angiography 2 version 1.9.10.0; Heidelberg Engineering, Germany) was used for FAF. Images were captured from the posterior field, aiming to delimit the estimated area of the preserved retina (without atrophy) with the caliper available in the device’s software, also evaluating the FAF of the macular region, the presence of a hyper-FAF ring, and the presence of hypo-FAF peripheral pigments.

### OCT

Heidelberg Spectralis SD-OCT version 6.16.8 (Heidelberg Engineering, Germany) was used. To assess the central foveal thickness, the Early Treatment Diabetic Retinopathy Study, volume map was produced from a dense scan. To assess the presence of cysts/cystoid macular edema, epiretinal membrane and whether the ellipsoid zone (EZ) was intact, disorganized, or absent, vertical and horizontal line scans were performed. To evaluate the EZ, participants were classified based on the state of EZ, which could be absent (loss of the ellipsoid line), disorganized (when a large part of the integrity of the line was not preserved), or intact (when the line was preserved).

### Genetic analysis

All patients had previous molecular genetic testing using next-generation sequencing for inherited retinal diseases panels, carried out by Invitae, Mendelics, and Molecular Vision laboratories that included 224 and 333 genes associated with retinal dystrophies. Molecular tests were collected by saliva swab (oral mucosa) or blood sample. Segregation analysis was confirmed when possible. The variants found in the *EYS* gene were compared to those listed in ClinVar to identify their pathogenic status, following the American College of Medical Genetics (ACMG) guidelines^(13)^.

### Statistical analysis

Data were tabulated and analyzed using Microsoft Excel 2013 and RStudio version 4.2.3 (2023-03-15 ucrt). Data were analyzed descriptively and statistically. The mean ± standard deviation (SD) were obtained for symmetrical variables.

## RESULTS

### Clinicodemographic data

Twenty-two subjects were included in this study. Demographic, ophthalmic, and clinical features are summarized in table 1. Of this group, 68% (n=15) were female and aged between 18 and 64 years.

**Table 1:** Analysis of retrospective data from patients with RP-*EYS* (n=22)

Age (years)	Min-Max	Average ± SD
	18-64	43.91 ± 13.04
Total	n	(n%)
**Sex**		
Female	15	68
Male	7	32
**Age of symptom onset**		
1st decade (0-10 years)	4	18
2nd decade (11-20 years)	9	41
3rd decade (21-30 years)	3	14
≥4th decade (≥40 years)	5	23
**Lens status**		
Cataract and/or previous cataract surgery in at least one eye	12	55
Without cataract	10	45

* n= number of subjects; Min= minimum; Max= maximum.

Regarding the age of onset of RP symptoms, 41% (n=9) referred to the second decade. Regarding the first symptoms, 91% (n=20) claimed nyctalopia, 55% (n=12) claimed loss of visual field, 45% (n=10) claimed photophobia, and 27% (n=6) said they also had other symptoms, such as low VA, photopsia, glare, and changes in color vision. Fifty-five percent (n=12) of the subjects had cataracts and/or had undergone cataract surgery in at least one eye (Table 1), 91% (n=20) had some refractive error, and 73% (n=16) had myopic astigmatism.

### Clinical features

#### VA

VA ranged from 1.60 to 0.00 logMAR; the mean ± SD was 0.66 ± 0.53 logMAR.

#### SVF

**Figure 1:**
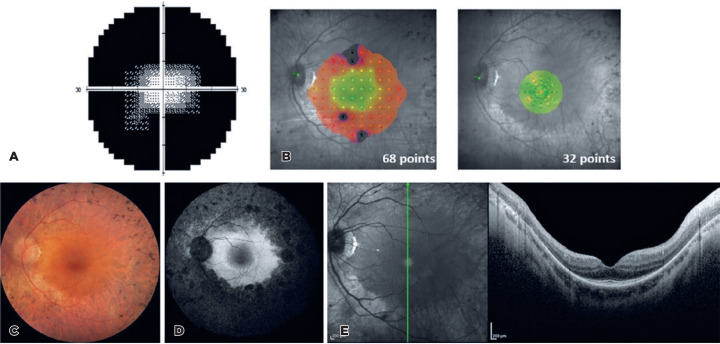
SVF, MP, FP, FAF, and OCT examinations of the left eye of subject no. 20. The images of this subject were chosen because they represent the main findings found in most patients with RP-*EYS*. (A) SVF: a grayscale graph that shows a tubular visual field. (B) MP: retinal sensitivity map made from evaluating 68 and 32 points on the retina. Maps show greater preservation of retinal sensitivity in the central region. (C) FP: image of the posterior pole of the fundus, where the presence of pallor of the optic nerve, bone spicules, RPE rarefaction, and vascular attenuation can be observed. (D) FAF: posterior pole image in which hypo-FAF can be observed in the macular region, a hyper-FAF ring close to the macular region, and a mottled pattern from the midperiphery. (E) OCT: vertical section image to evaluate the presence of cysts/cystoid macular edema, epiretinal membrane, and integrity of EZ.

**Figure 2:**
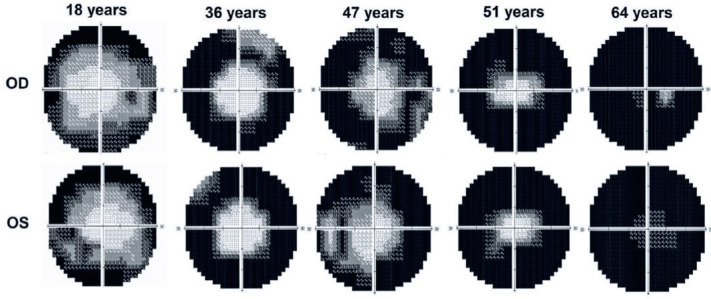
Grayscale graphs show static perimetry results at different ages.

In SVF, it was impossible to examine one patient due to extremely low vision. In another patient, the test results showed high false-positive and fixation loss rates. The visual field had a cloverleaf pattern, which was considered unreliable, as grayscale graphics in this pattern indicate tiredness or inattention; therefore, it was not considered reliable to enter the analyses. For the analysis, 20 subjects (40 eyes) were considered. The average foveal sensitivity threshold in the dB of the sample was 26.25 ± 8.67. During the evaluation of the grayscale graphs, 55% (n=22 eyes) had a tubular pattern on the visual field, 40% (n=16 eyes) had a total loss of visual field, and 5% (n=2 eyes) presented an arcuate scotoma. Therefore, most samples presented a tubular pattern visual field, as seen in figure 1A. Figure 2 shows the visual field narrowing as the patient ages, and the figure was assembled from examinations of patients who best represented disease progression, demonstrating the examination results in younger to older patients. In some cases, patients (even younger) presented greater visual field alteration than older patients.

### MP

Forty-one eyes were analyzed in 68 center points and 39 eyes in 32 center points. The average was 6.61 ± 8.11 dB in the 68 center points and 14.66 ± 9.62 in the 32 center points. A better result was observed in the 32-point test, where stimuli were projected in a more central region of the retina (Figure 1B). In some cases, it was impossible to perform all strategies in both eyes of all patients due to the difficult fixation.

### FP

Eighty percent (n=35 eyes) had optic disc pallor, 86% (n=38 eyes) had bone spicules in the midperiphery of the retina, and all (n=44 eyes) had abnormal retinal pigment epithelium (RPE) pigmentation and vascular attenuation. All these clinical findings can be observed in Figure 1C.

### FAF

Forty eyes underwent FAF. In two patients, it was impossible to perform the examination, possibly due to the advanced stage of the disease, which prevented the capture of images. Eighty-five percent (n=34 eyes) of the assessed eyes had hypo-FAF in the macular region, 30% (n=12 eyes) showed disorganization of the macular autofluorescence pattern (parts with hypo-FAF and parts with hyper-FAF), 70% (n=28 eyes) had a visible hyper-FAF ring, and 90% (n=36 eyes) showed hypo-FAF peripheral pigments and a mottled pattern. The main findings of the examination can be seen in Figure 1D. The estimated retinal area preserved (mean ± SD, mm^2^) was 36.13 ± 21.33. The central retina (macular region) was preserved, consistent with SVF and MP results, which showed greater retinal sensitivity in this region.

### SD-OCT

The mean ± SD foveal central thickness of 44 eyes was 246 ± 59.16 µm. The horizontal and vertical scans were analyzed. Figure 1E shows an image in the vertical section. Cystoid macular edema was classified regardless of severity. Therefore, both patients with small cysts and clinically significant edema were included in the analysis. Thus, 50% (n=22 eyes) had cystoid macular edema. The epiretinal membrane was noted in 23% (n=10 eyes) of the sample. Sixty-four percent (n=28 eyes) had disorganized EZ, and 36% (n=16 eyes) had absent EZ. OCT findings also integrated with the findings of functional examinations (SVF and MP) and the FAF, as a large part of the sample showed greater preservation of retinal layers in the central region of the retina close to the macula.

#### Genetic features

Twenty-six distinct variants were identified in the cohort. Genetic results revealed c.4957dupA(p.Ser1653Lysfs*2) and c.5928-2A>G (p.?) to be the two most prevalent variants, both present in 4 patients each. Table 2 shows the genotypes of patients in this study. Five of 22 patients cases were compound homozygotes (patients 2, 3, 16, 17, and 20), whereas the remaining were heterozygotes. Seven novel variants were identified: c.2055_2056delTG, c.2652dup, c.3213del, c.4651G>T, c.5182delA, c.7572G>A, and c.8245T>C, as highlighted in table 2. These variants had not been previously recorded in the scientific literature or databases.

**Table 2:** Genotype of patients with RP-*EYS*

ID	Sex	Nucleotide change	Protein change	Type of variant	Allele state	ACMG classification	Reference
1	M	Del exon 13-14	p.?	Deletion	HET	P	Pieras 2011
		c.7810C>T	p.Arg2604Cys	Missense	HET	VUS	Abdel 2010
2	F	**c.7572G>A**	p.Trp2524^*^	Nonsense	HOMO	P	NA
3	F	c.5928-2A>G	p.?	Splicing	HOMO	P	Pozo 2011
4	F	c.5928-2A>G	p.?	Splicing	HOMO	P	Pozo 2011
		Del exon 32-33	p.?	Deletion	HET	P	Audo 2010
5	M	c.32dup	p.Met12Aspfs^*^14	Frameshift	HET	P	Abu-Safieh 2013
		c.3443+1G>A	p.?	Splicing	HET	P	Abdel 2010
6	M	c.8779T>C	p.Cys2927Arg	Missense	HET	P	Jespersgaard 2019
		**c.3213del**	p.Thr1072Leufs^*^18	Frameshift	HET	P	NA
7	M	**2652dup**	p.Lys885^*^	Nonsense	HET	P	NA
		c.8834G>A	p.Gly2945Glu	Missense	HET	VUS	Barragán 2010
8	F	c.4120C>T	p.Arg1374^*^	Nonsense	HET	P	Barragán 2010
		c.5928-2A>G	p.?	Splicing	HET	P	Pozo 2011
9	F	**c.4651G>T**	p.Glu1551^*^	Nonsense	HET	P	NA
		**c.7572G>A**	p.Trp2524^*^	Nonsense	HET	P	NA
10	F	c.4957dupA	p.Ser1653Lysfs^*^2	Frameshift	HET	P	Iwanami 2012
		c.2528G>A	p.Gly843Glu	Missense	HET	P	Iwanami 2012
11	F	**c.2055_2056delTG**	p.Ala686Phefs^*^12	Frameshift	HET	P	NA
		c.3443+1G>A	p.?	Splicing	HET	P	Abdel 2010
12	F	c.5928-2A>G	p.?	Splicing	HET	P	Pozo 2011
		Del exon 24	p.?	Deletion	HET	P	
13	F	c.6557G>A	p.Gly2186Glu	Missense	HET	P	Abdel 2010
		c.4957dupA	p.Ser1653Lysfs^*^2	Frameshift	HET	P	Iwanami 2012
14	F	c.6557G>A	p.Gly2186Glu	Missense	HET	P	Abdel 2010
		c.4957dupA	p.Ser1653Lysfs^*^2	Frameshift	HET	P	Iwanami 2012
15	F	c.6794delC	p.Pro2265Glnfs^*^46	Frameshift	HET	P	Audo 2010
		Del exon 13-19	p.?	Deletion	HET	P	
16	M	c.6794delC	p.Pro2265Glnfs^*^46	Frameshift	HOMO	P	Audo 2010
17	F	c.6794delC	p.Pro2265Glnfs^*^46	Frameshift	HOMO	P	Audo 2010
18	F	**c.5182delA**	p.Ser1728Valfs^*^21	Frameshift	HET	P	NA
		c.6794delC	p.Pro2265Glnfs^*^46	Frameshift	HET	P	Audo 2010
19	F	**c.8245T>C**	p.Cys2749Arg	Missense	HET	VUS	NA
		Deletion exon 32-35	p.?	Duplication	HET	P	
20	M	c.9286_9295del	p.Val3096Leufs^*^28	Frameshift	HOMO	P	Beryozkin 2014
21	M	c.2528G>A	p.Gly843Glu	Missense	HET	P	Iwanami 2012
		c.7394C>G	p.Thr2465Ser	Missense	HET	LP	Hosono 2012
22	F	c.32dup	p.Met12Aspfs^*^14	Frameshift	HET	P	Abu-Safieh 2013
		Deletion exon 14-22	p.?	Copy number = +3	HET	P	

F= female; M= male; HET= heterozygous; HOMO= homozygous; NA= not available; VUS= variant of unknown significance; LP= likely pathogenic; P= pathogenic.Novel variants are highlighted in bold.

## DISCUSSION

According to Novais et al., multimodal imaging is essential to modern ophthalmic practice, has become the gold standard for clinical investigation, and is critical to successfully managing retinal disorders^(14)^. In general, this modality is considered fundamental, given the greatness of the findings in the examinations, which allow a better understanding of the clinical features of RP related to the *EYS* gene. By analyzing the samples, the integration of morphological findings with the function of the structures was achieved. The tubular visual field and MP showing greater functional sensitivity in the macular region corresponded to FAF, FP, and OCT images that showed preservation of the central retinal area.

Regarding demographic features, in the sample studied, most participants reported symptom onset in the second decade of life (11-20 years), consistent with findings in other studies, as they reported RP-*EYS* symptoms beginning between 16 and 21 years^(15^-^18)^. Nyctalopia and progressive visual field constriction were the main symptoms initially felt by the patients. In a study on Italian individuals with RP-*EYS*, Mucciolo et al. described these symptoms as the main complaint at disease onset^(19)^.

When analyzing the values of the visual field pattern in SVF, the prevalence of tubular visual field or total field loss (patients with the disease at a more advanced stage) was found. When evaluating MP results, patients presented better results in the 32 central retinal points than in the 68-point assessment. Marques et al. stated that the disease predominantly affects the rods, with nyctalopia and narrowing of the visual field being the most striking symptoms, and central vision is usually preserved until the end course of the disease^(20)^. Thus, in evaluating 32 points in MP, patients presented better retinal sensitivity values due to the test being performed in the most central area of the retina with greater functional preservation of vision.

The fundus findings of this sample were similar to several studies, as the main fundus features were rarefaction of RPE, bone spicules in the midperiphery, and vascular attenuation^(3^,^19^,^21^,^22)^. In FAF, most individuals had normal hypo-FAF patterns in the macular region, indicating preservation of this region. Furthermore, many had a hyper-FAF ring in the macular region, and almost all had hypo-FAF dots in the midperiphery retina and a mottled pattern. FAF images of all patients in this study followed the typical morphological pattern. According to Lo et al., this pattern is defined by the degeneration of the photoreceptor or RPE, initially occurring in the midperiphery and later moving pericentrally^(11)^. In the study of Suto et al. on Japanese patients with mutations in the *EYS* gene, OCT images showed a marked reduction in retinal thickness resulting from the loss of photoreceptor layers, a finding similar to this study^(21)^. Regarding the presence of small cysts or even clinically significant cysts and epiretinal membrane detected on OCT images, these findings were consistent with those found in the study of Mucciolo et al.^(19)^. According to Smith et al., EZ appears on the OCT image as a hyperreflective layer in every normal macula^(23)^. Retinal dystrophies can cause progressive deterioration of EZ as the photoreceptor layer degrades^(24)^. In the sample of this study, most subjects presented EZ disorganization, a state in which the interruption of EZ begins at the periphery and advances centrally as the island of intact photoreceptors erodes. These results were consistent with previous studies, as more preservation of the retinal layers only in the central retina was observed in OCT examinations^(17^,^19^,^25)^.

This study had some limitations during its development. A few patients had a part of examinations excluded due to low vision and nonreliable tests and another due to opacities. Furthermore, it was not possible to analyze the electrophysiological full-field ERG test. This examination was essential to study the electrical responses to visual stimuli generated by retinal cells. Future studies must evaluate patients with RP associated with the *EYS* gene using the electrophysiological test.

The age of the youngest participant in this study and several studies that evaluated RP-*EYS* was ~18 years old, and participants younger than this age were not identified, a fact that can be justified by the late clinical manifestation of the disease. Therefore, improving the molecular diagnosis and ophthalmological examinations can lead to the early discovery of RP-*EYS*.

This study enhanced the understanding of RP associated with the *EYS* gene. When analyzing examinations, patterns similarly manifested in a large part of the sample of individuals of different ages were identified. Therefore, RP related to the *EYS* gene presents a pattern in its manifestation, starting in adolescence with mild progression and advancing with age. Cataracts, cystoid macular edema, and epiretinal membrane were frequently found in these patients. By reporting novel variants, this study broadened the genetic spectrum of RP-*EYS*. The findings had crucial implications for future longitudinal studies and clinical trials, serving as a fundamental resource to be used as a basis for treatment trials for RP-*EYS*. Furthermore, this study will help classify variants and the genetic counseling process.

In summary, when analyzing this rare disease cohort, it is possible to understand the clinical features of RP related to the *EYS* gene through a multimodal analysis. These findings enhance the understanding of RP-*EYS* features, characterizing the spectrum of mutations of this gene in the Brazilian population.
